# Monte Carlo simulations of geometric deformation of Harrison–Anderson–Mick applicators used in intraoperative radiation therapy

**DOI:** 10.1002/acm2.70386

**Published:** 2025-11-27

**Authors:** Benjamin Insley, Brett Bocian, Sam Beddar, Reza Reiazi, Patrick James Jensen, Rachael M. Martin‐Paulpeter, Joshua Scott Niedzielski, David Flint, Gabriel Oliveira Sawakuchi, Luis Augusto Perles

**Affiliations:** ^1^ Empyrean Medical Systems, Inc Boca Raton Florida USA; ^2^ University of Miami Miami Florida USA; ^3^ Department of Radiation Physics The University of Texas MD Anderson Cancer Center Houston Texas USA; ^4^ The University of Texas MD Anderson Cancer Center UTHealth Houston Graduate School of Biomedical Sciences Houston Texas USA; ^5^ Accuray Inc. Sunnyvale California USA

**Keywords:** brachytherapy, HAM applicators, IORT, Monte Carlo simulation, treatment planning

## Abstract

**Background:**

The Harrison–Anderson–Mick (HAM) applicator is a high‐dose‐rate intraoperative radiotherapy (HDR‐IORT) applicator used to position Ir‐192 brachytherapy sources over surgically‐accessed tumor volumes or post‐resection tumor beds. Because of the lack of a 3D imaging system, dwell times are optimized pre‐surgery by a TG‐43–based treatment planning system (TPS) that assumes a perfectly flat applicator surface surrounded by an infinite water phantom. These assumed conditions are disparate from typical treatment conditions, especially in the pelvic regions, which often involve uneven patient surfaces and superficial irradiations with little to no backscatter material.

**Purpose:**

Develop and validate a Monte Carlo (MC) model of HDR‐IORT treatment with a HAM applicator and use this validated model to quantify inaccuracies in the dose calculations due to the simplified conditions assumed in the planning process.

**Methods:**

The Oncentra Brachy TPS was used to optimize dwell times and calculate the delivered relative dose distribution for a 6‐catheter, 8‐cm HAM applicator with 0.5‐cm dwell steps and 1.0‐cm catheter spacing. A TOPAS (v.3.9) MC model of this treatment was then developed and validated against the TPS dose calculations. Once validated, the TOPAS applicator model was modified to calculate the difference in doses due to changes in backscatter conditions, the incorporation of the actual applicator materials, and curvature of the applicator. Dose distributions were characterized using dose to the prescription point, percent depth doses, and two‐dimensional isodose curves.

**Results:**

Validation between the MC and TPS calculations was successful within 1.5% over a depth of 5.0 cm in water. Negligible dose differences were calculated when assuming the applicator is made completely of water versus modeling all the individual applicator components. Conversely, semi‐infinite phantom geometry had more than 5% loss in dose to the prescription point due to the absence of backscatter. Most severely, curvature radii in the range of 5.4 cm (shallow curvature) to 0.9 cm (steep curvature) had dose differences of 5%–25%, regardless of whether the curvature was along the catheter length or transverse to it.

**Conclusions:**

This study quantified the changes in dose due to material and geometric differences that are currently not accounted for by the TPS. While not accounting for the material of the applicator contributes to negligibly, the presence of backscattering material can contribute up to 5%. The radius of the bending of the applicator was found to potentially have the largest impact on the dosimetry of the central plane, with deviations up to 25%.

## 1 INTRODUCTION

Intraoperative radiotherapy (IORT), or the application of radiation during surgical procedures, is commonly employed in the control of surgically‐exposed tumor volumes and post‐resection tumor beds.^1^ Because these procedures involve physically exposing the target and bringing the radiation source in close proximity to it, clinicians typically employ localized, low‐penetration radiotherapy modalities. IORT is performed with several different modalities, including megavoltage electron therapy (IOERT), low‐energy/orthovoltage x‐ray therapy (IOXRT), and high dose rate brachytherapy (HDR‐IORT).^1^, ^2^, ^3^ Our institution performs HDR‐IORT for select primary advanced and locally recurrent colorectal cancers as well as for select head and neck cases. From 2014 to 2024, 371 patients at University of Texas MD Anderson Cancer Center received HDR‐IORT treatment using catheters embedded in a flexible silicone flap known as Harrison–Anderson–Mick (HAM) applicator. Approximately 93% of the IORT cases were primary or recurrent colorectal patients, with treatment sites predominantly located in the pelvic side wall (58%) and sacrum (30%). In a 10‐year retrospective study conducted at our institution, 5‐year local control rates were 94% and 56% for primary and recurrent tumors, respectively. Overall survival rates for the same cohort were 61% and 56% for primary and recurrent cases, respectively.^1^, ^4^, ^5^, ^6^

There is, however, inter‐ and intra‐institutional variability in the outcomes of rectal IORT treatments with the literature reporting an overall survival range of 35% to 79% and local control range of 57% to 100% for locally advanced tumors,^7^, ^8^, ^9^ which leads to questions concerning IORT efficacy. Although some variation can be attributed to different methodologies such as varying surgical margins, a wide range of dose prescriptions, the use of chemotherapy agents, or use of IOERT versus HDR‐IORT, a previous study from our institution used their TG‐43‐based treatment planning system to uncover dosimetric inaccuracies related to the conformation of the HAM applicators around uneven target surfaces.^2^ The present study seeks to update and extend the conclusions of this study with the following objectives:

1. Develop and validate a detailed Monte Carlo (MC) model of an HDR‐IORT treatment with a HAM applicator
2. Use the validated MC model to apply material and geometric perturbations, and quantitatively summarize the calculated dose errors compared to the idealized treatment template

## 2 MATERIALS AND METHODS

**FIGURE 1:**
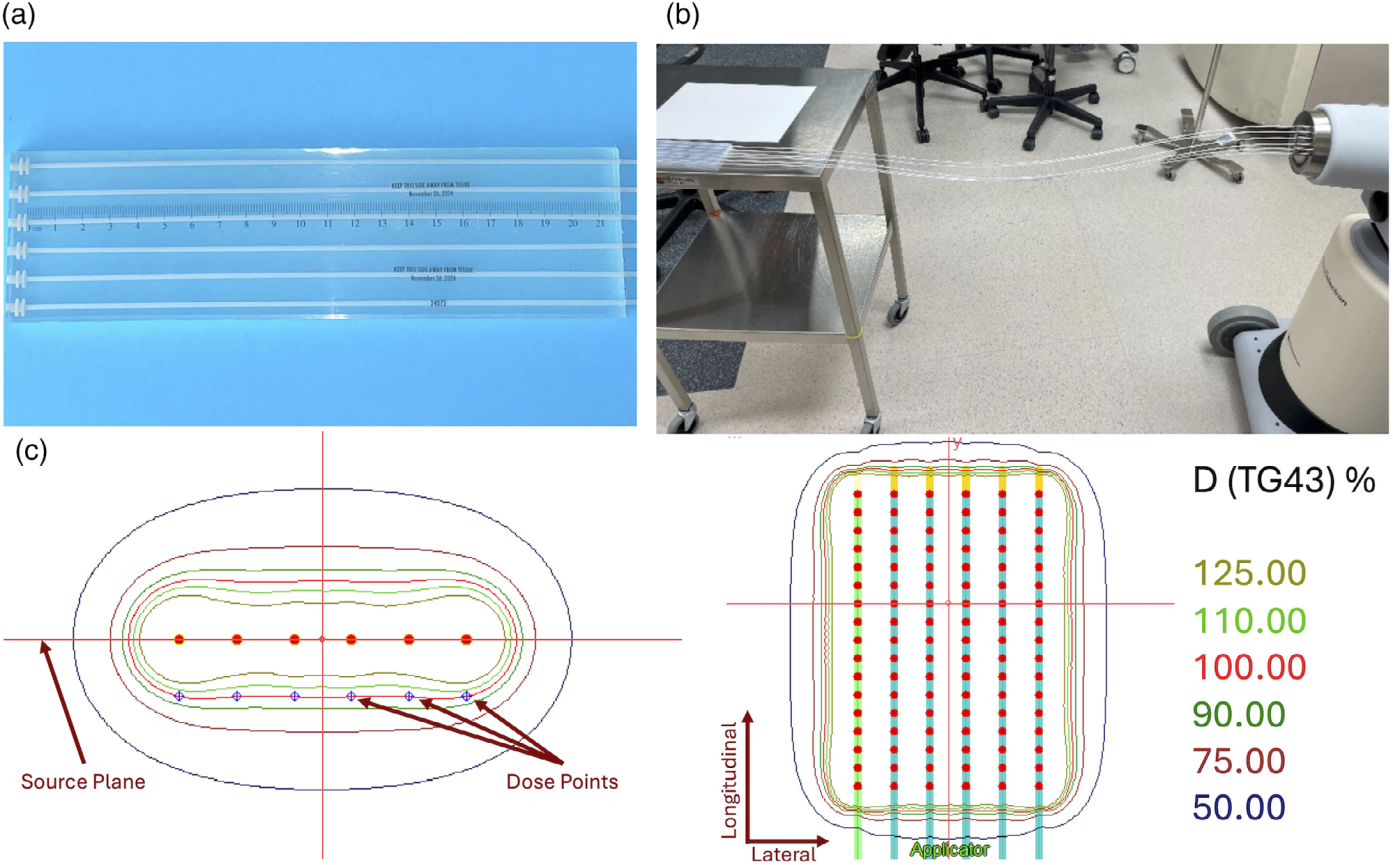
HDR‐IORT with a HAM applicator. a) HAM applicators. A single size is shown: 6‐catheter. b) Afterloader connection showing how an HDR afterloader connects to the HAM catheters to move sources in and out of the applicator. c) Oncentra treatment planning system showing a 6‐catheter, 8‐cm HAM applicator. The left and right panels display the axial and coronal perspectives, respectively, of the dwell positions in the TPS. The coronal view looks down onto the applicator, matching the perspective in Figure 1a. The lateral and longitudinal directions are defined in the coronal view, with longitudinal along the length of the catheters and lateral perpendicular to this across the catheters. The axial view defines the source plane as the lateral–longitudinal plane crossing through the dwell positions (red dots). The axial view also shows blue dots corresponding to dose calculation points. The dose calculated at these points is used to optimize the dwell times.

The HAM applicator (Mick Radio‐Nuclear Instruments, Inc., NY, USA) features a 0.8‐cm‐thick silicone flap interspersed with plastic catheters 1.0 cm apart. HAM applicators are manufactured with various lengths and numbers of catheters to accommodate target regions of various sizes (Figure 1a). A remote afterloader is used to guide an Ir‐192 source through the catheters at pre‐planned dwell positions in 0.5‐cm steps (Figure 1b), with optimized dwell times to produce a homogeneous dose 1.0‐cm from the catheter plane based on the prescribed dose and seed strength. We currently use a Nucletron microSelectron v3 (Nucletron, Elekta AB, Stockholm, Sweden) to deliver the IORT treatments. Treatment planning is done in Oncentra Brachy treatment planning system (TPS) version 4.6.0 (Nucletron, Elekta AB, Stockholm, Sweden) using the TG‐43 approach.^10^ Because these pelvic surgeries are long, we select the plans from a library of HAM applicators containing a range of different numbers of catheters. The templates represent the catheters in a flat geometry in an infinite water volume (Figure 1c). These simplifications ignore the following common non‐reference conditions: 1) non‐water materials are present within the applicator, including a silicone flap (C_2_H_6_OSi; density 1.09 g cm^−3^), high density polyethylene (HDPE) catheters (C_2_H_4_; density 0.97 g cm^−3^), and air gaps within the catheters (the exact polymer for the catheter material was not found in the literature, so HDPE was chosen as a near‐water‐equivalent plastic whose exact definition should not affect the results significantly in multi‐dwell point treatment plans^11^); 2) patients comprise non‐infinite water geometry, resulting in disparate scatter conditions compared to the TG‐43 assumptions, especially in superficial exposures with little to no backscatter material placed on top of the applicator; and 3) applicators are bent to match the target's topology, shifting the dwell points’ relative locations. Each of these conditions can cause the actual delivered dose to differ from the planned dose.

We restricted our analysis to the 6‐catheter HAM applicator with an 8‐cm‐long treatment region (6‐catheter, 8‐cm), whose dimensions represent the median size of applicators available at UT MD Anderson CC. Although the manufacturer provides a wide variety of applicator sizes, our institution stocks applicators with 4, 5, 6, 9, and 12 catheters, each with a maximum treatment length of 22 cm.

The plans generated for the 6‐catheter, 8‐cm HAM applicator were optimized in the Oncentra Brachy TPS to achieve a uniform dose 1.0 cm below the source plane, defined in Figure 1c, at points directly below each dwell position. The central axis dose 1.0 cm from the source plane was considered the 100% dose for normalization. Dose to water was calculated according to TG‐43 by Oncentra Brachy in a 0.1‐cm dose grid starting from the surface of the applicator (0.5 cm from the source plane) to a depth of 5.0 cm from the source plane, extending ±5.5 and ±7.0 cm in the lateral and longitudinal directions, respectively. The TPS used the inverse planning simulated annealing algorithm^12^ to optimize the dwell times across 102 total dwell positions—that is 17 dwells in each of the 6 catheters, placed in 0.5‐cm increments along the 8‐cm total treatment length.

**FIGURE 2:**
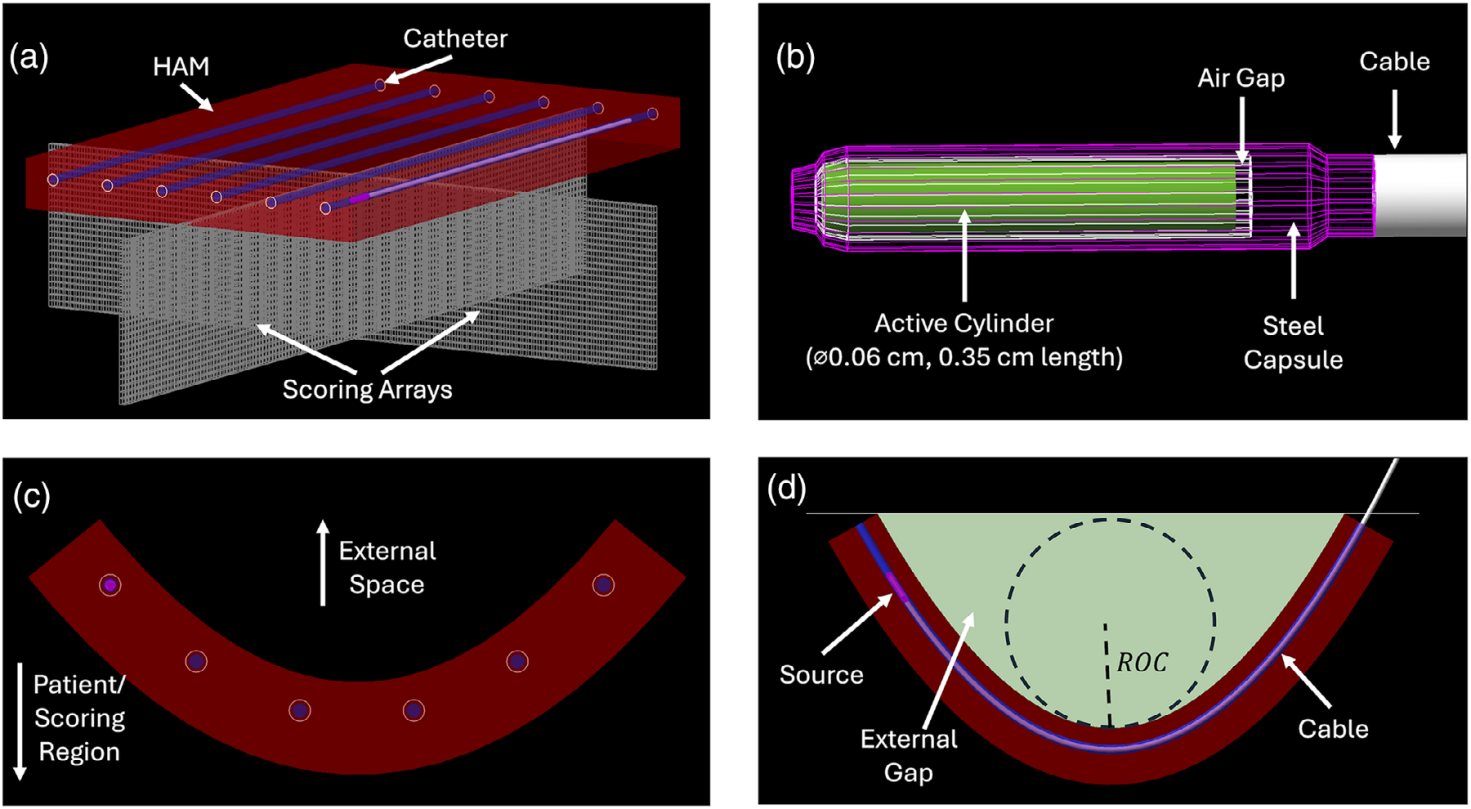
MC simulation geometry. a) Flat applicator. The HAM applicator is shown as the red block, the catheters and internal air gaps are shown as the blue cylinders within the applicator, the source and its cable are shown inside the nearest catheter, and the 0.1‐cm voxel scoring arrays are shown below the applicator. The simulation is situated within a 100 × 100 × 100 cm water volume. b) Microselectron v3 Ir‐192 source geometry, per manufacturer specifications.^15^ c) HAM applicator bent laterally. The bending occurs away from the patient for both lateral and longitudinal directions. All curved applicators were created using CAD software and imported into TOPAS. d) HAM applicator bent longitudinally. The external gap not included in 2c shows where air is included in the semi‐infinite phantom simulations. In those simulations, everything within the external gap and above is set to air. Everything else outside the applicator is water. In the infinite phantom simulations, everything outside the applicator is water. This image also displays the radius of curvature (ROC).

The accompanying MC model was developed in TOPAS (v.3.9, GEANT4 v.10.07p03, release date November 19, 2021)^13^, ^14^ and is displayed in Figure 2. The model features an Ir‐192 microSelectron v3 source designed according to manufacturer specifications.^13^, ^14^, ^15^, ^16^ During a simulation, the source is translated to each dwell position within the applicator, where it emits a number of photon histories proportional to the dwell time calculated in the corresponding treatment plan. The initial position of each photon history was randomly sampled from a uniform distribution over the volume of the active cylinder (Figure 2b), and the momentum directions were sampled isotropically.^17^ The energy of each photon history was sampled from a discrete spectrum acquired from Rivard et al.^18^ and validated with TOPAS by Berumen et al.^16^ against TG‐43′s reported dose calculation parameters.^10^

The first simulation, displayed in Figure 2a, was used to validate our TOPAS model against the Oncentra Brachy calculations. To match the TG‐43 formalism employed by Oncentra Brachy, the simulation omitted all applicator components including the silicone flap, HDPE catheters, and the catheters’ internal air gaps. Only the source and its cable were included, and the entire simulation was situated inside a 100‐cm cubical water environment to approximate the infinite water assumption of TG‐43.^10^ Dose was scored along the central lateral and longitudinal planes of the source arrangement in 0.1‐cm cubical voxels to match the TPS dose grid. Given the low effective energy of the Ir‐192 source and the relatively coarse resolution of the scoring grid, TOPAS's track length estimator was employed to approximate dose to water as collisional KERMA. This method of dose approximation was validated for our microSelectron v3 TOPAS model in 2021 by Berumen et al. also using 0.1‐cm voxels.^16^ The percentage depth dose (PDD) and 2D isodose curves calculated in these simulations were compared with those calculated by the TPS. All dose maps were normalized to the mean dose value for voxels ±0.5 cm from the central axis, 1.0 cm from the source plane.

Following this validation procedure, we progressively added the non‐water materials present in a typical treatment but not represented in the TPS model. First, we converted the simulation from an infinite water geometry to a semi‐infinite geometry. In other words, one side of the applicator (upstream of the surface 0.3 cm from the source plane) was replaced with air to represent superficial IORT treatment where no material is placed on top of the applicator. Next, the non‐water materials of the applicator itself were included for both the infinite and semi‐infinite simulations. In all the simulations described in this paragraph, the applicator was assumed to be perfectly flat, allowing us to isolate the effects of the phantom model currently in the TPS. The dose distributions were compared using the PDDs, lateral and longitudinal plane isodose curves, and prescription dose point values. The prescription dose point values were calculated as the mean dose 1.0 cm from the source plane toward the patient, averaged over the voxels ±0.5 cm from the central axis. All dose maps were normalized by the TPS simulation's prescription dose, thereby allowing the direct comparison of all these dose maps to that calculated by the TPS.

The final set of simulations analyzed the dose differences for curved applicators (Figure 2c,d), following the curvatures applied by Beddar et al.^2^ Lateral and longitudinal curvatures were implemented using the quadratic function:

(1) $$y=ax^{2}$$

where y is distance along the depth axis and x is distance along either the lateral or longitudinal axis of the applicator. Tumor beds are not guaranteed to be parabolic, but this simple method allows for easy parameterization and was previously discussed and recommended over other functions.^2^ The curvature parameter, a, was set to the same values used by Beddar et al.: 0.09, 0.25, and 0.54 cm^−1^. This represents the full range of curvatures experienced in a clinical setting, from flat (a=0) to very severe ($a=0.54\;cm^{-1}$). These values correspond to radii of curvature of approximately 5.4, 2.8, and 0.9 cm, respectively.^2^ Radius of curvature was calculated as the radius of the circle with the same first and second derivatives as the origin of our parabolas (Figure 2d). An explanation of how new dwell positions were calculated and how the scoring grid was deformed to match the applicator curvature is provided in the Supporting Information. This scoring grid deformation is an especially critical part of the analysis because it transforms the coordinates of the bent applicator simulations to look like the flat applicator simulations—i.e., this scoring grid deformation maintains consistent distances between the scoring voxels and the applicator no matter the applied curvature (mimicking a target region conforming to the applicator surface).

As in the other simulations, dose maps were normalized by the TPS dose calculations and compared using lateral and longitudinal plane isodose curves and prescription dose values. Each bent applicator simulation was repeated four times for the various material conditions (infinite vs. semi‐infinite phantom geometry, and all‐water vs. full applicator materials).

All simulations were run using the G4EmStandardPhysics_option3 physics list with a 0.05‐mm range cut. Since the track length estimator relies only on the energy fluence of photons and tabulated mass energy absorption coefficients, electron tracking is unnecessary and was deprecated to increase the simulation efficiency.^16^ Each simulation was run for a total of $9.0018\times 10^{8}$ histories across all 102 dwell positions, and history‐by‐history standard deviations were calculated for each voxel. Simulations were carried out on the Seadragon High Performance Computing Cluster at UT MD Anderson CC using 28 parallel CPUs per simulation. CPU clock speeds among nodes in the cluster with a minimum speed of 1.9 GHz. Simulation times varied between 2 and 11 h, depending on the scoring complexity of the simulation and the node it was assigned.

## 3 RESULTS

**FIGURE 3:**
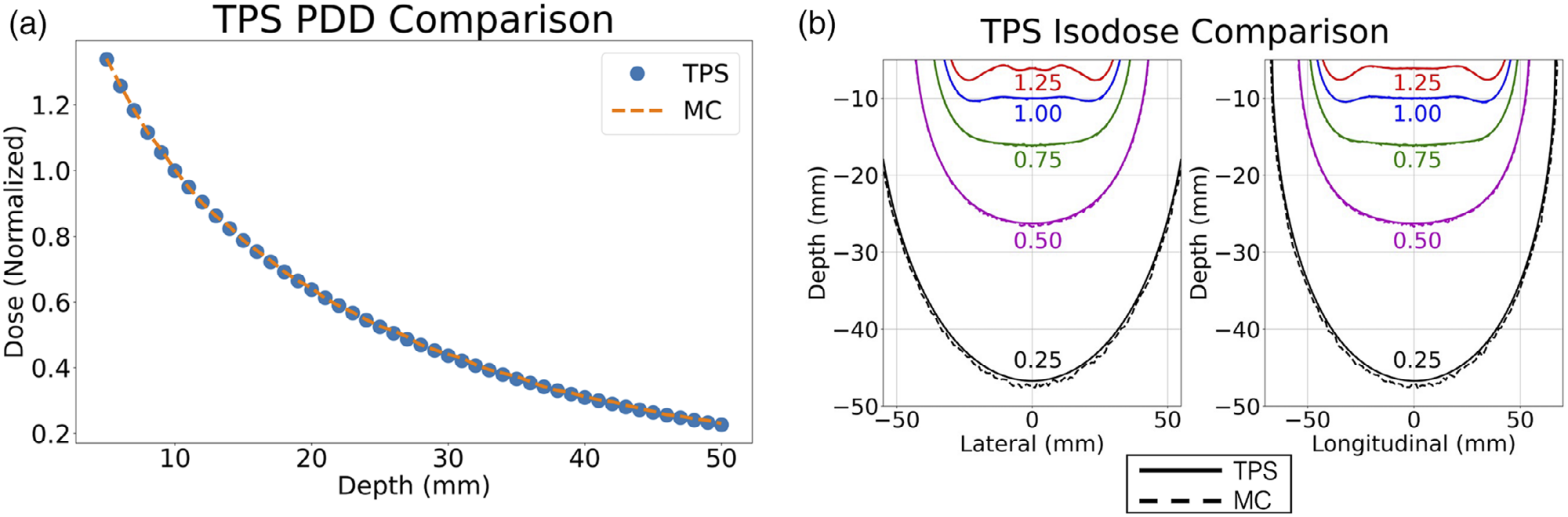
Model validation. a) PDD comparison. b) Lateral and longitudinal isodose comparison. TPS and MC curves are difficult to distinguish visually except near the 0.25 isodose due to the high quality of the agreement.

Figure 3a,b display the PDDs and lateral and longitudinal isodose curves, respectively, of the dose distributions calculated by the TPS and those calculated with our MC code for the TG‐43–based treatment model (infinite water phantom, no applicator materials present, and flat applicator). The MC dose distribution has a maximum relative standard error of 0.90%, and the agreement between MC and TPS is within 1.50% across both the lateral and longitudinal dose maps to a depth of 5 cm. Doses were normalized to the mean of the voxels 1.0 cm from the source plane (Depth=−1.0cm), ±0.5 cm from the applicator's central depth axis.

When the full applicator materials (silicone, HDPE, and air gaps) were implemented into this simulation for the infinite and semi‐infinite phantom geometries, negligible dose differences (0.4% and 0.3%, respectively) were calculated compared to the TPS all‐water assumption. Even adjusting the applicator density ±5% to account for manufacturing uncertainties only changed these dose differences by ∓0.2%. The same level of equivalence was calculated in the bent applicator simulations, displayed later. Given that the water‐equivalence of the HAM applicator materials for HDR radiotherapy has been confirmed previously,^11^ the rest of this results section and discussion will focus on the dose perturbations of infinite vs. semi‐infinite phantom geometry and applicator curvature. Each of the following figures still incorporates full applicator materials for accurate dose calculations, but differences between water and applicator materials will not be highlighted further.

**FIGURE 4:**
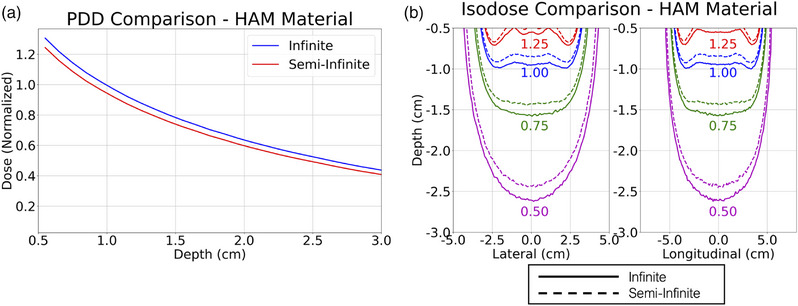
Flat applicator material comparisons. a) PDD comparison. b) Lateral and longitudinal isodose comparison. Full applicator materials are included in the data shown.

Figure 4 shows the MC‐calculated PDDs and lateral and longitudinal isodose lines for the flat applicator dose distributions with either infinite or semi‐infinite phantom geometry. As stated in the Methods, all doses were normalized by the TPS validation simulation's prescription dose to allow direct comparisons. Prescription dose values were calculated as the mean of the same set of voxels used to normalize the TPS validation simulation (1.0 cm from the source plane, ±0.5 cm from the source central depth axis); these prescription doses are 99.6% for infinite phantom geometry and 94.4% for semi‐infinite phantom geometry. These plots have been limited to 3‐cm depth to highlight the clinically relevant region and help zoom in on subtle isodose differences.

**FIGURE 5:**
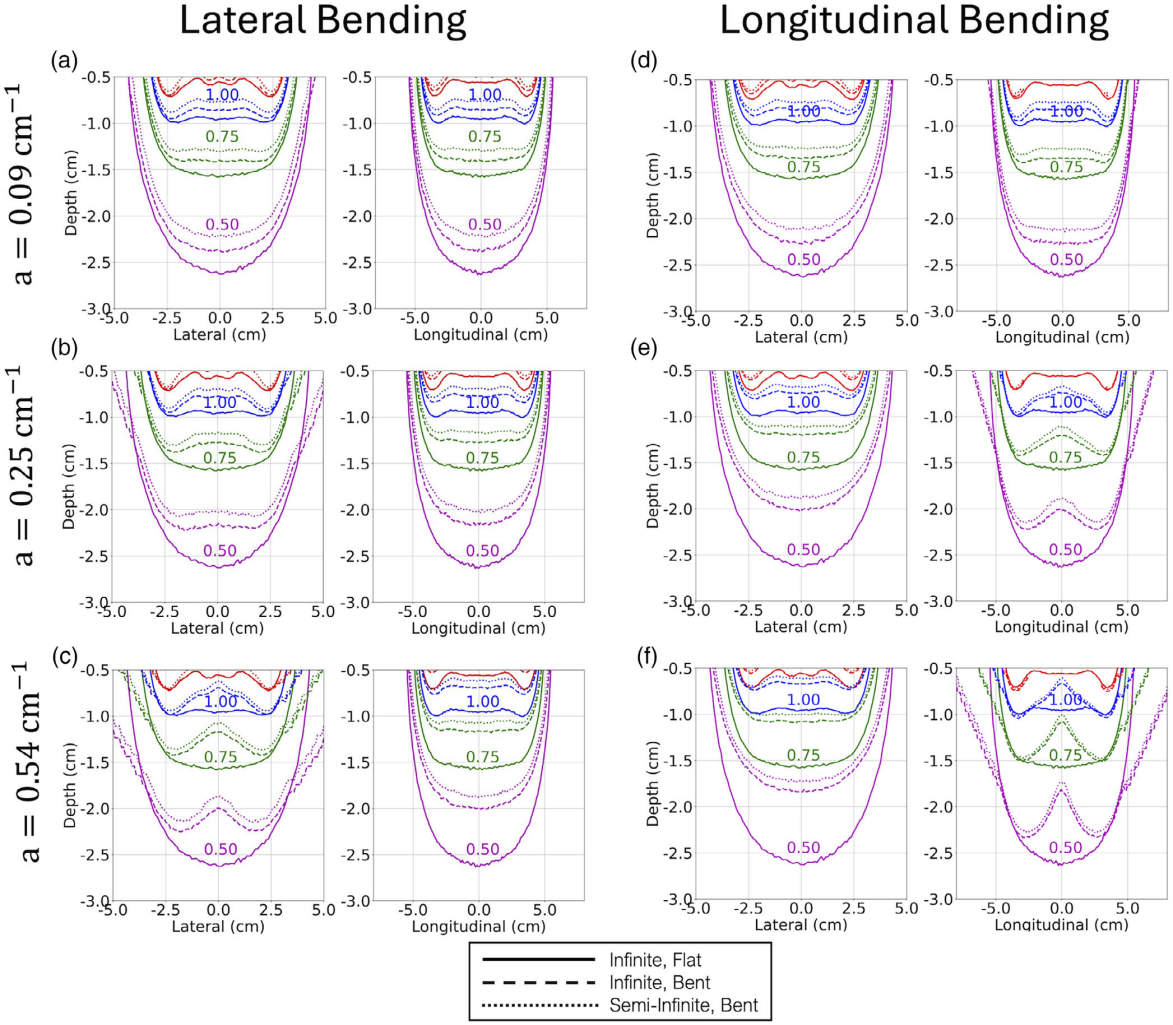
Bent applicator lateral and longitudinal isodose curves. a) Lateral bending, $a=0.09\;cm^{-1}$. b) Lateral bending, $a=0.25\;cm^{-1}$. c) Lateral bending, $a=0.54\;cm^{-1}$. d) Longitudinal bending, $a=0.09\;cm^{-1}$. e) Longitudinal bending, $a=0.25\;cm^{-1}$. f) Longitudinal bending, $a=0.54\;cm^{-1}$. Full applicator materials are included in the data shown.

**FIGURE 6:**
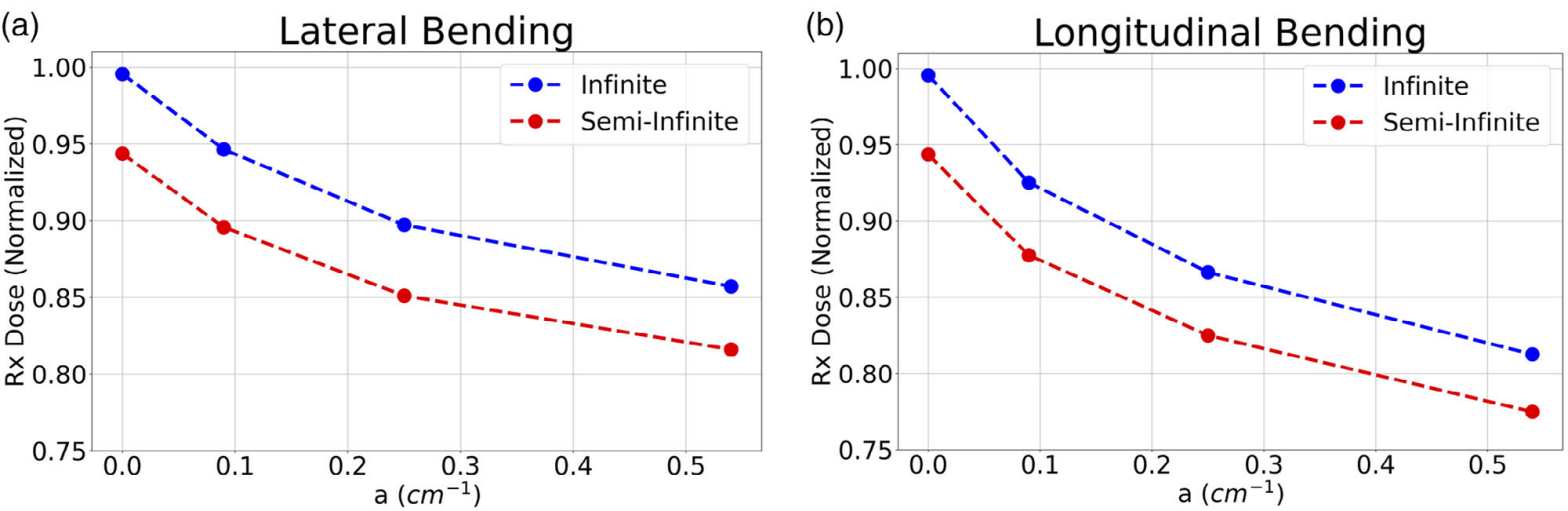
Prescription dose comparison. Full applicator materials are included in the data shown.

Finally, Figure 5 displays the isodose curves for the lateral and longitudinal bending simulations in their transformed coordinates, for both infinite and semi‐infinite phantoms. Each plot also contains the TPS dose distribution for comparison (labeled ‘Infinite, Flat’). Prescription doses as a function of the bending parameter (a) for these simulations are shown in Figure 6. The same normalization value was once again used for these plots to allow for direct quantitative comparison to the TPS calculations, and prescription dose is defined using the same voxels mentioned previously. Again, the coordinates of these isodose plots were transformed according to the derivation in Supporting Information. Failing to transform these coordinates to their flat applicator counterparts would preclude direct isodose comparisons. These plots have also been limited to 3‐cm depth to highlight the clinically relevant region and help zoom in on subtle isodose differences.

## 4 DISCUSSION

The first step of this study was to design and validate a TOPAS model of an IORT treatment with a 6‐catheter, 8‐cm HAM applicator. Figure 3 demonstrates that this model has sufficient statistical power (0.90% maximum standard deviation) and sufficient accuracy compared to the commissioned TPS (1.50% maximum error) to draw significant conclusions from the dose deviations seen in Figures 4, 5, 6.

Figure 4 examines the dose differences between the infinite phantom assumption of TG‐43 and the semi‐infinite phantom geometry often used in an HDR‐IORT treatment. The presence of backscattering material had noticeable effects on the dose distribution, which can be seen in all the graphs of Figure 4. The difference in the prescription dose between infinite and semi‐infinite phantom geometry is 5.2%, highlighting the statistically significant contribution of backscattered photons from material on the opposite side of the applicator. These differences are also noticeable in the semi‐infinite simulation's lowered PDD and shallower isodose curves. The isodose curves, however, seem to roughly retain their shape, indicating that the correct dose can be delivered across much of the dose map by simply scaling the dwell times by a constant factor. Thus, a plan would only need to be scaled, and not reoptimized, to fix this discrepancy. In an actual treatment, the amount of backscattering material is somewhere between the infinite and semi‐infinite scenarios. Oftentimes, wet surgical packing is placed on top of the applicator to keep the applicator in place, which can provide some backscatter material. Therefore, 5.2% should be seen as an upper bound on the dosimetric effect of non‐infinite phantom geometry, and wet surgical packing should be used whenever possible to minimize dose loss.

Much larger dose differences were observed when the applicator was bent, either laterally or longitudinally. Figure 5 demonstrates how the isodose lines in the non‐bending direction (i.e., the longitudinal plane for the lateral bending and, vice versa, the lateral plane for the longitudinal bending) progressively shifts the isodose lines toward the surface as the curvature increases. The isodose curves in the bending direction, however, not only become shallower but also change shape and become highly peaked toward the central plane. This is mostly due to the applicator's quadratic curvature, where the peripheral dwell positions move further than the central dwell positions as the applicator bends. So, while it seems the isodose curves in the non‐bending direction can be compensated with a simple rescaling of the dwell times, the isodose lines in the bending direction would require dwell time reoptimization to recover their original shape.

As a final note on the dose differences due to applicator bending, Figure 6 summarizes the prescription dose loss as a function of the curvature parameter (a). The ranges of dose loss are similar between lateral and longitudinal bending (5%–25%). The longitudinal bending doses are slightly lower because 1) the curved cable intercepts the radiation field, providing additional attenuation, and 2) the selected treatment length (8 cm) is longer than the lateral length, exacerbating the dose loss from the furthest dwell positions. Overall, in comparison to the dose differences observed in Figure 4, these are certainly large errors that go uncorrected in clinical practice.

Although this study focused on the 6‐catheter, 8‐cm, we expect other applicator sizes to follow a similar general trend in dose discrepancies. Future work is needed to accurately quantify the dose differences across applicator sizes and various lengths as a function of the orientation and radius of curvature. We also acknowledge that some smaller applicators like 4‐ and 5‐catheters do not have the same flexibility as the larger ones and are less likely to bend laterally.

To optimize the applicator treatment and compensate for its curvature, an individualized approach is required, such as utilizing three‐dimensional (3D) imaging technologies or developing an applicator library that encompasses a range of different curvatures. The imaging options currently available include CT‐on‐wheels and C‐Arm Cone Beam CT (CBCT). Both are available in our surgery department but are not currently integrated with our TPS. Other obstacles to implementing such technologies include limited access to the surgical area, since these are complex surgeries requiring a large amount of instrumentation, and time constraints precluding reconstruction of multiple catheters. Building a library of applicators with various pre‐determined curvatures is also feasible and fits with current clinical workflow. Although using such a library is practical, the necessary number of discreet curvatures per number of catheters has yet to be determined as well as a reliable method to measure it. Further enhancements to the treatment planning system can include a more accurate dose calculation engine such as collapsed cone convolution or Monte Carlo methods.^19^, ^20^

As stated in the introduction, the observed variability in the outcomes of IORT in colorectal surgeries is likely related to the differences in the methodology, however we believe that our study can help reduce uncertainties when comparing cross‐modality treatments such as IOERT and HDR‐IORT. The methodology developed in this study can be used to accurately calculate the dose when a 3D imaging system such as C‐Arm Cone Beam CT becomes available in the operating theater or, alternatively, in a retrospective study using post‐surgical CT images.

## 5 CONCLUSION

Because few centers offer HDR‐IORT treatment with a HAM applicator, it remains an understudied treatment modality. This study sought to build upon Beddar et al.’s 2006 work by developing and validating an MC model of the treatment and calculating differences due to material and geometric inaccuracies. We first demonstrated that our model matches the commissioned TPS's calculations within 1.5% for an ideal setup. Then, we found a 5% difference in delivered dose due to differences in backscattering material and up to 25% difference due to bending of the applicator in two different orientations. Overall, our findings show significant differences between planned and delivered doses may be present in IORT treatments with HAM applicators due to missing backscatter and the applicator curvature not being considered in the treatment plan.

## CONFLICT OF INTEREST STATEMENT

The authors declare no conflicts of interest.

## Supporting information



Supporting information
