# Supplementary material for: Monte Carlo simulations of geometric deformation of Harrison–Anderson–Mick applicators used in intraoperative radiation therapy
Source: J Appl Clin Med Phys. 2025 Nov 27;26(12):e70386. doi: 10.1002/acm2.70386 (PMC12658346; doi:10.1002/acm2.70386)
Supplement: Supplementary file 1 — Supporting information [file ACM2-26-e70386-s001.docx]

**Supplemental Materials**

To calculate the dwell positions of the bent applicators, we assumed that as the applicator bends along quadratic curves, the total length of the applicator along the source plane remains constant. This allows us to calculate the new dwell positions of the sources using the following formalism, diagrammed in Figure S1a.

First, consider the lateral or longitudinal distance between the origin of the applicator and any dwell position (we consider the lateral and longitudinal distances separately, but equivalently, since we only bend the applicators along one of the directions at a time). In the flat applicator, this distance, $t$, is equivalent to the pathlength of the line through each dwell. As the applicator bends about this origin, the pathlength from origin to any dwell should remain constant, assuming no lengthening or contraction of the silicone material or shifting of the catheters within the silicone. This pathlength constraint is formalized as:

|  | $t=\int_{0}^{x^{'}} \sqrt{1+\left( \frac{dy}{dx} \right)^{2}}dx=Const.$ | (2) |
| --- | --- | --- |

where $x^{'}$ is the new lateral or longitudinal position of a given dwell after bending the applicator along $y=ax^{2}$. The integrand can be expanded using Equation 1:

|  | $t=\int_{0}^{x^{'}} \sqrt{1+\left( 2ax \right)^{2}}dx=\frac{\ln\left( \sqrt{1+\left( 2ax^{'} \right)^{2}}+2ax^{'} \right)}{4a}+\frac{x^{'}\sqrt{1+\left( 2ax^{'} \right)^{2}}}{2}$ | (3) |
| --- | --- | --- |

In the case of the flat applicator, $t$ is equivalent to $x^{'}$, which is given by the TPS lateral and longitudinal dwell positions, displayed in the first columns of Tables S1 and S2 in Supplementary Materials. Because $t$ remains constant, these values can be plugged into Equation 3, allowing us to solve for $x^{'}$ implicitly using the Newton-Raphson Method^21^ for given values of $a$. The values of $x'$ for the lateral and longitudinal dwell positions are given in Tables S1 and S2, respectively. Note that during lateral bending (Figure 2c), only the lateral coordinates are transformed – the longitudinal coordinates remain unchanged – and vice versa for longitudinal bending (Figure 2d). Finally, the transformed depth coordinate can be calculated as $y^{'}=ax^{'2}$.

In addition to transforming the source location, longitudinal bending changes the local trajectory of the source. For these dwell positions, an additional angle is calculated to rotate the source about its lateral axis and match the direction of the catheter (Figure 2d). This angle, $\theta^{'}$, is calculated as the angle between the parabola’s tangent line and the longitudinal plane:

| $\theta^{'}=\arctan\left( \frac{dy}{dx} \right)=\arctan\left( 2ax^{'} \right)$ | (4) |
| --- | --- |

Values of $\theta$ for each level of curvature are displayed in Table S3 in Supplementary Materials.

In an actual treatment, the applicator is bent to conform to a non-flat target surface. Thus, simulations involving bent applicators should conform the scoring grid to the applicator curvature. This allows direct comparison between the dose distribution of the flat applicator and that of the bent applicators. The most efficient means of implementing this conformal scoring grid is by transforming the coordinates of a Cartesian one. This process is diagrammed in Figure S1b and formalized as follows.

First, consider the relationship between the source plane and scoring grid when the applicator is perfectly flat. For a given voxel at Cartesian coordinates $\left( x_{G},y_{G} \right)$, the nearest point on the flat applicator’s source plane is at the same $x$-coordinate, $x_{G}$, and a distance $d=|y_{G}|$ above the voxel. The vector pointing between these two points (the voxel and the nearest point on the source plane) is perpendicular to the source plane. As the applicator is bent, a given voxel’s coordinates in the untransformed grid, $\left( x_{G}^{'},y_{G}^{'} \right)$, are related back to the flat applicator’s scoring grid coordinates, $\left( x_{G},y_{G} \right)$, using these two principles: 1) its perpendicular distance (i.e., its shortest distance) to the curved source plane, $d$, gives the voxel’s transformed $y$-coordinate, and 2) the pathlength from the origin to the nearest point on the curved source plane gives the voxel’s transformed $x$-coordinate.

In practice, we start with the untransformed coordinates of the voxels in a Cartesian scoring grid. The square of the distance between a voxel located at $\left( x_{G}^{'},y_{G}^{'} \right)$ and a point on the source plane parabola, $\left( x^{'},y^{'} \right)$, is given by:

| $d^{2}=\left( x^{'}-x_{G}^{'} \right)^{2}+\left( y^{'}-y_{G}^{'} \right)^{2}=\left( x^{'}-x_{G}^{'} \right)^{2}+\left( a{x^{'}}^{2}-y_{G}^{'} \right)^{2}$ | (5) |
| --- | --- |

The point on the source plane parabola nearest to this voxel is found by minimizing equation 5 by setting its derivative with respect to $x^{'}$ equal to 0:

| $\frac{d\left( d^{2} \right)}{dx^{'}}=0$ | (6) |
| --- | --- |

Plugging Equation 5 into Equation 6 and rearranging gives:

| ${x'}^{3}+\frac{\left( 1-2ay_{G}^{'} \right)}{2a^{2}}x'-\frac{x_{G}^{'}}{2a^{2}}=0$ | (7) |
| --- | --- |

This is a cubic equation of the form:

| $u^{3}+pu+q=0$ | (8) |
| --- | --- |

with:

| $p=\frac{\left( 1-2ay_{G}^{'} \right)}{2a^{2}}$ | (9) |
| --- | --- |
| $q=-\frac{x_{G}^{'}}{2a^{2}}$ | (10) |

If $a$ is only positive and $y_{G}^{'}$ is defined to be negative below the applicator (within the patient), then the quantity $\frac{q^{2}}{4}+\frac{p^{3}}{27}$ is strictly positive, and Cardano’s formula states there is one real root given by $\sqrt[3]{u_{1}}+\sqrt[3]{u_{2}}$, where:

| $u_{1}=-\frac{q}{2}+\sqrt{\frac{q^{2}}{4}+\frac{p^{3}}{27}}$ | (11) |
| --- | --- |
| $u_{2}=-\frac{q}{2}-\sqrt{\frac{q^{2}}{4}+\frac{p^{3}}{27}}$ | (12) |

Once $x^{'}$ is determined from Equations 7 – 12, $y^{'}$ is calculated as $y^{'}=ax^{'2}$, and the distance, $d$, is given by Equation 5. In addition, the pathlength along the source plane, $t$, corresponding to $x^{'}$ is given by Equation 3. Thus, the voxel located at coordinates $\left( x_{G}^{'},y_{G}^{'} \right)$ in the bent applicator geometry corresponds to the voxel at coordinates $\left( t,-d \right)$ in the flat applicator geometry.

To implement this, the coordinates of the bent applicator scoring arrays were first transformed to their flat applicator counterparts. Then, we iterated through the flat applicator scorers’ coordinates and, for each set of flat applicator voxel coordinates, located a nearest neighbor in the bent applicator scorers using their transformed coordinates. Then, a 5x5 voxel window was created around the nearest neighbor and used to interpolate to the desired coordinates. This interpolation was handled by SciPy’s RBFInterpolator (v.1.11.4), which performs radial basis function interpolation using an inverse quadratic basis function. This interpolation was compared to simple nearest neighbor interpolation to ensure accuracy.


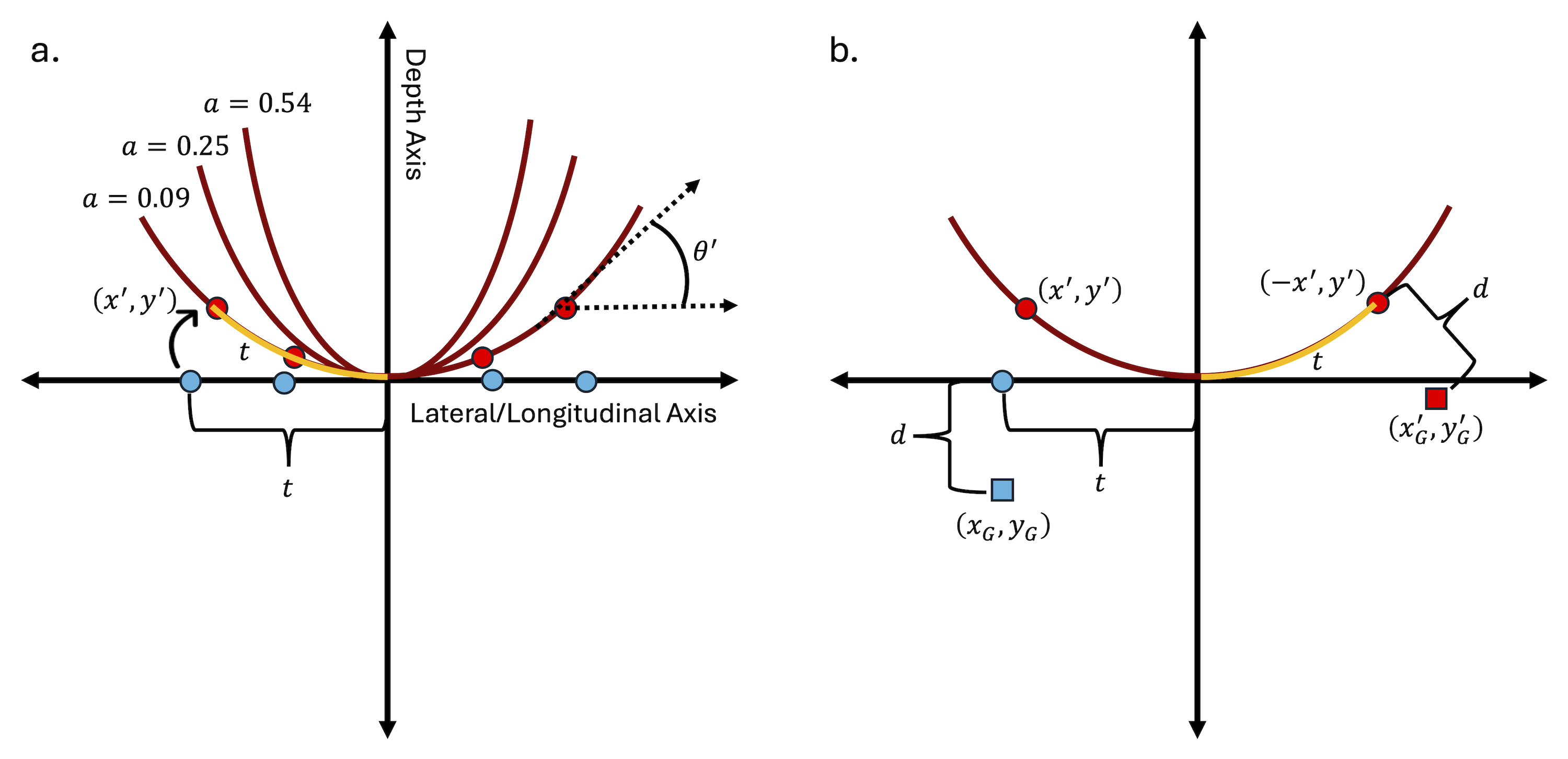


Figure S1: Deformation geometry. a) Dwell position transformation. The horizontal axis represents either the lateral or longitudinal direction of the applicator, and the vertical axis points into/away from the patient along the depth direction. Light blue circles represent the original dwell positions and red circles represent the corresponding dwell positions in the bent applicator. The maroon arcs show the curvature of the applicator along the source plane according to the quadratic function $y=ax^{2}$ for $a=0.09, 0.25,$ and 0.54 $cm^{-1}$. The distance between the axis origin and the furthest dwell position in the flat applicator is given by $t$. This distance is equivalent to the pathlength shown by the yellow curve from origin to furthest dwell position in the bent applicator. $\theta^{'}$ is shown on the right side of the curve as the angle between the maroon curve’s tangent at that point and the horizontal axis. b) Scoring grid transformation. The light blue square represents a voxel position in the flat applicator scoring array, and the light blue circle shows a corresponding position on the source plane with the same horizontal position. The red circle and square show the transformed version of the light blue circle and square as the applicator is bent. Note that the red circle and square are moved to the opposite side of the vertical axis for clarity. The perpendicular distance between circle and square, d, and pathlength from origin to circle, t, are conserved.

Table S1: Lateral dwell positions. Note that only the positive values are shown. Negative $t$ values are accompanied by negative $x^{'}$ values.

|  | $\vert x^{'}\vert\left( cm \right)$ | | | |
| --- | --- | --- | --- | --- |
| $\vert t\vert$ (cm) | Flat applicator | $a=0.09 cm^{-1}$ | $a=0.25 cm^{-1}$ | $a=0.54 cm^{-1}$ |
| $0.5$ | $0.5$ | $0.499$ | $0.495$ | $0.479$ |
| $1.5$ | $1.5$ | $1.483$ | $1.394$ | $1.212$ |
| $2.5$ | $2.5$ | $2.425$ | $2.142$ | $1.742$ |

Table S2: Longitudinal dwell positions. Note that only the positive values are shown. Negative $t$ values are accompanied by negative $x^{'}$ values.

|  | $\left\vert x^{'} \right\vert\left( cm \right)$ | | | |
| --- | --- | --- | --- | --- |
| $\vert t\vert$ (cm) | Flat applicator | $a=0.09 cm^{-1}$ | $a=0.25 cm^{-1}$ | $a=0.54 cm^{-1}$ |
| $0.0$ | $0.0$ | $0.0$ | $0.0$ | $0.0$ |
| $0.5$ | $0.5$ | $0.499$ | $0.495$ | $0.479$ |
| $1.0$ | $1.0$ | $0.995$ | $0.964$ | $0.881$ |
| $1.5$ | $1.5$ | $1.483$ | $1.394$ | $1.212$ |
| $2.0$ | $2.0$ | $1.960$ | $1.785$ | $1.494$ |
| $2.5$ | $2.5$ | $2.425$ | $2.142$ | $1.742$ |
| $3.0$ | $3.0$ | $2.876$ | $2.470$ | $1.966$ |
| $3.5$ | $3.5$ | $3.313$ | $2.773$ | $2.170$ |
| $4.0$ | $4.0$ | $3.735$ | $3.056$ | $2.359$ |

Table S3: Longitudinal rotations. Note that only the positive values are shown. Negative $t$ values are accompanied by negative $\theta^{'}$ values.

|  | $\left\vert\theta^{'} \right\vert\left( ^{\circ} \right)$ | | | |
| --- | --- | --- | --- | --- |
| $\vert t\vert$ (cm) | Flat applicator | $a=0.09 cm^{-1}$ | $a=0.25 cm^{-1}$ | $a=0.54 cm^{-1}$ |
| $0.0$ | $0$ | $0.00$ | $0.00$ | $0.00$ |
| $0.5$ | $0$ | $5.14$ | $13.90$ | $27.37$ |
| $1.0$ | $0$ | $10.15$ | $25.73$ | $43.58$ |
| $1.5$ | $0$ | $14.94$ | $34.88$ | $52.62$ |
| $2.0$ | $0$ | $19.43$ | $41.75$ | $58.21$ |
| $2.5$ | $0$ | $23.58$ | $46.96$ | $62.01$ |
| $3.0$ | $0$ | $27.37$ | $51.00$ | $64.78$ |
| $3.5$ | $0$ | $30.81$ | $54.20$ | $66.89$ |
| $4.0$ | $0$ | $33.91$ | $56.79$ | $68.57$ |
